# A phase II study of biweekly oxaliplatin plus infusional 5-fluorouracil and folinic acid (FOLFOX-4) as first-line treatment of advanced gastric cancer patients

**DOI:** 10.1038/sj.bjc.6602573

**Published:** 2005-04-26

**Authors:** F De Vita, M Orditura, E Matano, R Bianco, C Carlomagno, S Infusino, V Damiano, E Simeone, M R Diadema, E Lieto, P Castellano, S Pepe, S De Placido, G Galizia, N Di Martino, F Ciardiello, G Catalano, A R Bianco

**Affiliations:** 1 Division of Medical Oncology, ‘F Magrassi & A Lanzara’ Department of Clinical and Experimental Medicine, Second University of Naples School of Medicine, Naples, Italy; 2 Division of Medical Oncology, Department of Molecular and Clinical Endocrinology and Oncology, ‘Federico II’ University of Naples School of Medicine, Naples, Italy; 3 II Division of General Surgery, ‘F Magrassi & A Lanzara’ Department of Clinical and Experimental Medicine, Second University of Naples School of Medicine, Naples, Italy; 4 VII Division of General Surgery, ‘F Magrassi & A Lanzara’ Department of Clinical and Experimental Medicine, Second University of Naples School of Medicine, Naples, Italy

**Keywords:** advanced gastric cancer, chemotherapy, 5-fluorouracil, oxaliplatin

## Abstract

The aim of the study was to assess the toxicity and the clinical activity of biweekly oxaliplatin in combination with infusional 5-fluorouracil (5-FU) and folinic acid (FA) administered every 2 weeks (FOLFOX-4 regimen) in patients with advanced gastric cancer (AGC). A total of 61 previously untreated AGC patients were treated with oxaliplatin 85 mg m^−2^ on day 1, FA 200 mg m^−2^ as a 2 h infusion followed by bolus 5-FU 400 mg m^−2^ and a 22 h infusion of 5-FU 600 mg m^−2^, repeated for 2 consecutive days every 2 weeks. All patients were assessable for toxicity and response to treatment. Four (7%) complete responses and 19 partial responses were observed (overall response rate, 38%). Stable disease was observed in 22 (36%) patients, with progressive disease in the other six (10%) patients. Median time to progression (TTP) and median overall survival (OS) were 7.1 and 11.2 months, respectively. National Cancer Institute Common Toxicity Criteria grade 3 and 4 haematologic toxicities were neutropenia, anaemia and thrombocytopenia in 36, 10 and 5% of the patients, respectively. Grade 3 peripheral neuropathy was recorded in three (5%) patients. FOLFOX-4 is an active and well-tolerated chemotherapy. Response rate (RR), TTP and OS were comparable with those of other oxaliplatin-based regimens, suggesting a role for this combination in gastric cancer.

Despite a decreasing trend in its incidence, gastric cancer remains the second most common cause of cancer-related death (Parkin, 1998); furthermore, there was a change in the site of origin within the stomach, with a rising incidence of cancer of cardia and gastroesophageal junction (Blot *et al*, 1991). Advanced gastric cancer (AGC) remains incurable with a median survival of 6–9 months (Alberts *et al*, 2003). However, in randomised trials, chemotherapy was demonstrated to result in both a significant survival advantage and an improved quality of life when compared to best supportive care (Murad *et al*, 1993; Pyrhonen *et al*, 1995; Glimelius *et al*, 1997). 5-Fluorouracil (5-FU) remains the most extensively studied single agent, and continuous intravenous infusion is frequently used in combination chemotherapy regimens (Alberts *et al*, 2003). The association of epirubicin, cisplatin and 5-FU in continuous infusion, also known as ECF regimen, showed an impressive activity in a phase II trial, with 12% complete responses (CR). Compared with FAMTX in a phase III randomised trial, ECF obtained a higher response rate, a superior median time to progression (TTP) and a better overall survival (OS) (Webb *et al*, 1997). Because of these results, ECF was considered as the European standard treatment. Several platinum analogues have been developed with the aim of improving the efficacy and tolerability of cisplatin. Recently, oxaliplatin, a third-generation platinum compound with the 1,2-diaminocyclohexane (DACH) carrier ligand, has entered the clinical practice, showing a different toxicity profile, with neurotoxicity being the dose-limiting toxicity (Extra *et al*, 1990; Raymond *et al*, 1998). Oxaliplatin showed additive or synergistic activity when associated to 5-FU, even in 5-FU-resistant cell lines (Becouarn *et al*, 2001). It has been registered worldwide for the treatment of advanced colorectal cancer, where, in association with 5-FU, it was reported to yield a response rate of 36–58% (Levi *et al*, 1992; Levi *et al*, 1994; De Gramont *et al*, 2000). In *in vitro* studies, oxaliplatin was demonstrated to inhibit the growth of several gastric cancer cell lines (Eriguchi *et al*, 2003). In a phase II study involving previously CDDP-treated patients with AGC, a bimonthly association of oxaliplatin, 5-FU and leucovorin was associated with a 26% RR and an acceptable toxicity profile (Kim *et al*, 2003). Furthermore, in a phase II study, a biweekly FOLFOX-6 regimen (oxaliplatin 100 mg m^−2^, FA 400 mg m^−2^ followed by bolus 5-FU 400 mg m^−2^ and a 46 h continuous infusion of 5-FU 3000 mg m^−2^) was demonstrated to be an active and safe treatment in 51 chemotherapy-naive patients with AGC (Louvet *et al*, 2002). These results were recently extended by a multicentre phase II study in 41 AGC patients treated with a modified FOLFOX schedule (oxaliplatin 85 mg m^−2^, FA 500 mg m^−2^ followed by 5-FU 2600 mg m^−2^ as a 24 h continuous infusion every 2 weeks) (Al-Batran *et al*, 2004). Here, we report the results of a multicentre phase II trial aimed at determining the efficacy and safety of a FOLFOX-4 regimen as first-line treatment in 61 AGC patients.

## PATIENTS AND METHODS

### Eligibility

Patients with histologically proven unresectable locally advanced or metastatic gastric cancer were considered eligible for the study if they met all of the following criteria: measurable disease; cytologically or histologically proven single metastatic lesion as the only manifestation of the disease; aged >18 and <75 years; ECOG PS <2; life expectancy >3 months; adequate bone marrow, hepatic and renal function; no prior palliative chemotherapy; written informed consent before enrolment in the study. Previous adjuvant chemotherapy was allowed if more than 6 months had elapsed between the end of adjuvant therapy and first relapse.

### Treatment and toxicity assessment

Chemotherapy consisted of oxaliplatin 85 mg m^−2^ on day 1, FA 200 mg m^−2^ as a 2 h infusion followed by bolus 5-FU 400 mg m^−2^ and a 22 h infusion of 5-FU 600 mg m^−2^ on days 1 and 2 every 2 weeks. The use of central venous catheters and disposable pumps allowed chemotherapy administration on an outpatient basis. This regimen was administered until progression. Toxicity was assessed before starting and each 2-week cycle using the National Cancer Institute Common Toxicity Criteria (NCI-CTC), version 1.0, except neurotoxicity. Peripheral sensitive neuropathy was graded according to the following oxaliplatin-specific scale: grade 1, paresthesias/hypoesthesias of short duration with complete recovery before the next cycle; grade 2, paresthesias/hypoesthesias persisting between two cycles without functional impairment; grade 3, permanent paresthesias/hypoesthesias resulting in functional impairment (Caussanel *et al*, 1990). Treatment delays and dose modifications were based on the worst adverse effects observed during the previous cycle. Oxaliplatin was reduced to 75 mg m^−2^ in case of persistent (>14 days) paresthesia or temporary (7–14 day) painful paresthesia or functional impairment. In case of persistent (>14 days) painful paresthesia or functional impairment, oxaliplatin was omitted from the treatment until recovery. Together with reductions in the dose of oxaliplatin, the bolus dose of 5-FU was reduced to 300 mg m^−2^ and the infusion dose to 500 mg m^−2^ in the event of grade 3 or 4 neutropenia, thrombocytopenia, diarrhoea, stomatitis or other drug-related adverse effects of grade 3. Treatment was delayed by up to 3 weeks until the patient recovered from various adverse effects, the neutrophil count exceeded 1500 per cubic millimetre and the platelet count exceeded 100 000 per cubic millimetre. In the event of skin toxicity of grade 3 or 4, only the dose of 5-FU was reduced.

### Study end points

In 4 weeks before starting chemotherapy, all patients underwent the following studies: physical examination, complete blood cell count, hepatic and renal function tests, chest and abdominal CT scan and an ultrasound endoscopy. Physical examination, hepato-renal function tests and blood counts were performed every cycle. Tumour evaluation was assessed every three cycles according to WHO criteria. Complete response is defined as the disappearance of all known lesions and absence of new lesions; partial response (PR) as a reduction of 50% or more in the sum of the product of the two-dimensional measures of all known lesions and absence of new lesions; stable disease (SD) as a reduction of <50% or an increase <25% in the sum of the product of the two-dimensional measures of all known lesions and absence of new lesions; and progressive disease (PD) as an increase of >25% in the two-dimensional measures of one or more known lesions or as the appearance of at least one new lesion. Treatment was continued until disease progression or unacceptable toxicity occurred or until a patient chose to discontinue treatment. All patients who completed at least three cycles of chemotherapy were deemed assessable for response. All eligible patients were included in the response and survival analysis on an ‘intent-to-treat’ basis. The primary end point of the study was the overall response rate; secondary end points were toxicity, evaluation of TTP and OS.

### Statistical analysis

The two-stage minimax design for phase II trial of Simon (1989) was adopted, selecting an alpha error=0.05 and a beta error=0.20. The minimum activity required for this experimental treatment was 30%, while the alternative hypothesis was to obtain a 50% response rate. Therefore, the accrual had to be stopped if less than six responses were obtained with the first 16 patients. Otherwise, more than 18 responses among a total of 46 patients are required to accept this hypothesis.

Statistical analysis was carried out using the BMDP statistical package (BMDP Statistical Software Inc., Los Angeles, CA, USA). In all analyses, the significance level was specified as *P*<0.05. The Kaplan–Meier method was used to analyse TTP and OS.

## RESULTS

### Patient characteristics

A total of 61 patients were enrolled from March 2001 to June 2003. The characteristics of the patients are summarised in Table 1. The median age was 64 years and the majority of patients had a Performance Status 1 according to the ECOG scale. A G3 undifferentiated tumour was present in 31 patients (51%), while a G2 moderately differentiated tumour was observed in 28 patients (46%). Metastatic disease was present in the majority of the study population (56 out of 61 patients); liver (38 out of 61 patients, 62%) and lymph nodes (25 out of 61 patients, 36%) were the most common sites of metastases. The median number of organs involved was two (range, 1–4), with 27 patients (44%) having two organs involved, 10 patients (16%) having three organs involved and two patients (3%) having four organs involved. Prior surgery was performed in 37 out of 61 patients. No patient had received prior radiotherapy, while 10 patients had received adjuvant chemotherapy with 5-FU and folinic acid according to the Machover regimen following radical surgery.

### Tumour response

All 61 patients were evaluable for response to therapy. Major responses were observed in 23 patients (38%; 95% CI, 25.8–50.2), with four patients achieving a CR (7%) and 19 showing a PR (31%). One CR was pathologically confirmed, since the patient underwent curative surgery after nine cycles of chemotherapy and was alive without evidence of disease as of 31 December 2003. Stable disease was obtained in 22 additional patients (36%). Progressive disease was observed in 16 patients (26%). Therefore, the overall tumour growth control (CR+PR+SD) was 74% (45 out of 61 patients).

### Survival

**Figure 1:**
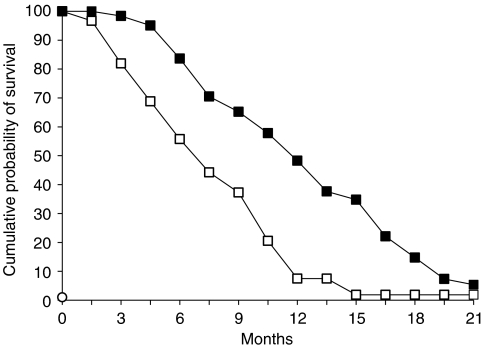
Estimated 21-month cumulative probability of survival and time to treatment progression in 61 AGC patients. Shown are TTP (□□□□□□) and OS (▪▪▪▪▪) in the 61 AGC patients treated with the FOLFOX-4 regimen.

**Figure 2:**
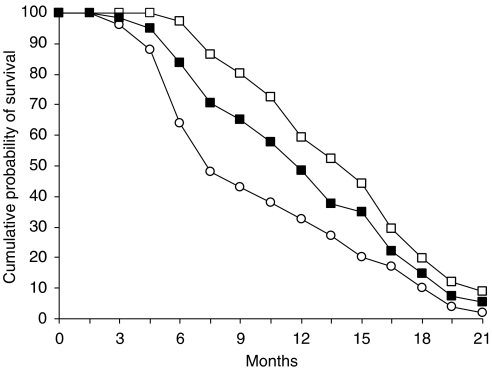
Estimated 21-month cumulative probability of survival in 61 AGC patients. Shown are OS in the 36 AGC patients treated with second-line chemotherapy (□□□□□□) and in the 25 patients treated with best supportive care (○○○○○) following disease progression. The figure also depicts OS in the 61 patients (▪▪▪▪▪).

All patients were included in the survival analysis on an intent-to-treat basis. The median follow-up was 11.6 months (range, 6.9–20.3 months). The median TTP was 7.1 months (95% CI, 5.6–8.7) (Figure 1). At the time of analysis (31 December 2003), 44 patients had died and 17 were alive. The median OS was 11.2 months (95% CI, 9.66–14.39). At the time of analysis, two patients who achieved a CR were disease free at 19.9 and 7.6 months of follow-up, respectively. Following documentation of disease progression, 36 out of 61 patients received a second-line docetaxel- or irinotecan-based chemotherapy. As shown in Figure 2, patients receiving a second-line treatment had a statistically significant (*P*=0.0026; long-rank test) longer median OS (12.7 months; 95% CI, 10.7–15.6) than patients (25 patients) receiving only best supportive care (median OS, 9.4 months; 95% CI, 5.6–12.7).

### Toxicity

A total of 450 cycles were administered, with a median of seven cycles for patients (range, 3–15 cycles). A total of 45 out of 61 patients (74%) received at least six cycles, 34 out of 61 patients (56%) received at least eight cycles and 14 out of 61 patients (23%) received at least 10 cycles. The occurrence and the incidence of main toxicities are reported in Table 2. The most common toxicities were haematologic. The National Cancer Institute Common Toxicity Criteria grade 3 and 4 neutropenia, leucopenia, anaemia and thrombocytopenia were recorded in 22 out of 61 (36%), 11 out of 61 (16%), six out of 61 (10%) and three out of 61 (5%) patients, respectively. Three out of 61 patients experienced febrile neutropenia. No NCI-CTC grade 4 gastrointestinal toxicity was observed, while grade 3 diarrhoea, nausea and vomiting were recorded in 5, 5 and 2% of the patients, respectively. Neurotoxicity was moderate and was observed in 30% (grade 1 in 11%, grade 2 in 14% and grade 3 in 5%) of the patients. The three patients who experienced a grade 3 neurotoxicity received a dose of oxaliplatin ranging from 935 to 1275 mg m^−2^ and cycles from 11 to 15. Three patients (5%) discontinued treatment because of treatment-related side effects, specifically neutropenia (one patient), diarrhoea (one patient) and neurotoxicity (one patient). No treatment-related death was reported.

## DISCUSSION

Although gastric cancer is considered a relatively chemotherapy-sensitive tumour with an overall response rate ranging between 30 and 60%, survival of AGC patients remains unsatisfactory, with a median survival time of 6–9 months (Ho *et al*, 1988; Kelsen *et al*, 2002). None of the current regimens can be considered as an optimal therapy for AGC and new therapeutic strategies are needed to achieve a better clinical efficacy with an acceptable toxicity profile. In the present study, we administered the combination of FA, 5-FU and oxaliplatin as first-line therapy to patients with AGC. In all, 23 of 61 (38%) patients achieved an objective response, with a 7% CR rate. The overall median TTP was 7.14 months, with a median OS of 11.2 months. The results of this study confirm the activity of a biweekly FOLFOX-4 regimen in the first-line treatment of AGC patients. To our knowledge, this is the largest phase II study in this patient population. Indeed, the other two phase II trials of oxaliplatin, 5-FU and FA combination in chemotherapy-naive AGC patients enrolled 54 (49 patients were assessable for response) and 41 (37 patients were assessable for response) patients, respectively (Louvet *et al*, 2002; Al-Batran *et al*, 2004). Table 3 summarises the results of our study in comparison with the two previously published phase II trials of oxaliplatin, FA and 5-FU as first-line therapy in AGC patients. RR was comparable in the three studies. However, the median TTP and OS in the present study were slightly better than those reported in the other two studies. The prolonged survival observed in our study cannot be related to a selected patient population with a good outcome, because 56 out of 61 (92%) patients had a metastatic disease and 63% had at least two metastatic sites. On the contrary, it could be explained by the fact that 59% of the study population received a second-line therapy. In fact, the 36 patients receiving a second-line treatment with docetaxel or irinotecan had a significant better median OS than the 25 patients receiving only best supportive care (12.7 *vs* 9.4 months; *P*=0.0026). These data suggest that salvage chemotherapy in AGC patients progressing after a first-line treatment may have a beneficial impact on survival, as has also been shown in other reports (Ajani *et al*, 2002; Giuliani *et al*, 2003). As previously observed in advanced colorectal cancer, in AGC patients, the FOLFOX regimen used in this study demonstrated an acceptable tolerability. Grade 3/4 neutropenia was the most common haematologic toxicity occurring in 36% of the patients, but febrile neutropenia was detected in only 3% of the patients. In a number of trials with oxaliplatin-based therapies, neurotoxicity was the most frequent side effect that led to treatment discontinuation. However, in our study, neurotoxicity was restricted to a limited number of patients. This may be due to a relatively low cumulative dose of oxaliplatin in our series, with a median number of seven cycles administered. In particular, in the study by Louvet *et al* (2002), in which oxaliplatin was administered at a dose of 100 mg m^−2^, peripheral neuropathy was reported in 87% of the treated population and was severe (grade 3 toxicity) in 21% of the patients. In our series, diarrhoea, nausea and vomiting were the most common side effects among nonhaematologic toxicities, but were mild and occurred less frequently when compared with CDDP-based regimens such as FUP and ECF (Kim *et al*, 1993; Webb *et al*, 1997). Similarly, alopecia was a rare side effect when compared with anthracyclines or etoposide-based regimens. The efficacy of this FOLFOX treatment was not different from that observed in phase II–III studies with other second- or third-generation polychemotherapy regimens in AGC (Lacave *et al*, 1991; Wils *et al*, 1991; Kelsen *et al*, 1992; Kim *et al* 1993; Rougier *et al*, 1994; Vanhoefer *et al*, 2000). Interestingly, the median TTP and OS observed in our study population are similar to those reported in a prospectively randomised study comparing ECF with the standard regimen FAMTX (Webb *et al*, 1997). In conclusion, FOLFOX-4 treatment appears to have a significant activity as first-line treatment for AGC patients, with an encouraging response rate and a mild toxicity profile; therefore, on the basis of these results, a phase III study comparing FOLFOX-4 *vs* ECF should be performed.

## Figures and Tables

**Table 1:** Patient characteristics

	**No. of patients (*n*=61**)	**%**
*Sex*		
Male	38	62
Female	23	38

*Age (years)*		
Median	64	
Range	47–75	

*Histologic diagnosis*		
Adenocarcinoma	55	90
Signet ring cell carcinoma	6	10

*Grading*		
1	2	3
2	28	46
3	31	51

*Performance status*		
0	9	15
1	39	64
2	13	21

*Adjuvant chemotherapy*		
Yes	10	16
No	51	84

*Disease status*		
Locally advanced	5	8
Metastatic	56	92

*No. of organs involved*		
1	22	36
2	27	44
3	10	16
>3	2	3

*Organs involved*		
Liver	38	
Lung	12	
Nodes	25	
Peritoneum	13	
Pleura	23	
Ovaries	1	
Pancreas	2	

**Table 2:** Main toxicities according to NCI-CTC scale

**Toxicity**	**Grade 1–2 (%)**	**Grade 3–4 (%)**
*Haematologic*		
Neutropenia	14 (23)	22 (36)
Leucopenia	11 (18)	12 (19)
Thrombocytopenia	20 (32)	3 (5)
Anaemia	23 (37)	6 (10)
Febrile neutropenia	—	2 (3)

*Gastrointestinal*		
Nausea	13 (21)	3 (5)
Vomiting	12 (19)	1 (2)
Diarrhoea	14 (23)	3 (5)
Stomatitis	7 (11)	1 (2)
Hepatic	1 (2)	—

Neurological^a^	16 (26)	3 (5)

*Others*		
Cutaneous	1 (2)	—
Alopecia	12 (19)	—
Asthenia	13 (21)	1 (2)
Allergic	2 (3)	—

a According to an oxaliplatin-specific scale (grade 0–3).

**Table 3:** Comparison of the published phase II studies using FOLFOX combinations as first-line chemotherapy in AGC patients

**Study**	**No. of evaluable patients**	**%RR (%CR)**	**Median TTP (mo)**	**Median OS (mo)**	**NCI-CTC G1–G2 toxicities (%)**	**NCI-CTC G3–G4 toxicities (%)**	**G1–G2 neurotoxicity (%)^a^**	**G3 neurotoxicity (%)^a^**
Louvet *et al* (2002)	49	45 (4)	6.2	8.6	Neutropenia (30)	Neutropenia (38)	66	21
					Leucopenia (43)	Leucopenia (19)		
					Anaemia (80)	Anaemia (11)		

Al-Batran *et al* (2004)	37	43 (3)	5.6	9.6	Anaemia (51)	Anaemia (7)	39	0
					Diarrhoea (37)	Diarrhoea (7)		
					Neutropenia (12)	Neutropenia (5)		

De Vita *et al* (2004) (this report)	61	38 (7)	7.14	11.2^b^	Neutropenia (23)	Neutropenia (36)	26	5
					Leucopenia (18)	Leucopenia (16)		
					Anaemia (37)	Anaemia (10)		

a According to an oxaliplatin-specific scale (grade 0–3).

b Overall survival was 12.74 months in 36 out of 61 patients receiving a second-line chemotherapy and 9.46 months in 25 out of 61 patients treated with best supportive care following disease progression.

AGC=advanced gastric cancer; CR=complete response; TTP=time to progression; OS=overall survival; mo=months.
